# Socio-demographic, environmental and behavioural risk factors of diarrhoea among under-five children in rural Ethiopia: further analysis of the 2016 Ethiopian demographic and health survey

**DOI:** 10.1186/s12887-020-02141-6

**Published:** 2020-05-20

**Authors:** Melkamu Molla Ferede

**Affiliations:** Department of Statistics, College of Natural and Computational Science, University of Gondar, Gondar, Ethiopia

**Keywords:** Diarrhoea, Risk factors, Under-five children, Rural Ethiopia

## Abstract

**Background:**

Diarrhoea is one of the major contributors to death among under-five children in Ethiopia. Studies conducted in different countries showed that rural children are more severely affected by diarrhoea than urban children. Thus, this study was aimed to identify the socio-demographic, environmental and behavioural associated risk factors of the occurrence of diarrhoea among under-five children in rural Ethiopia.

**Methods:**

Data for the study was drawn from the 2016 Ethiopian Demographic and Health Survey. A total of 8041 under-five children were included in the study. Binary logistic regression was used to assess the association of occurrence of diarrhoea with socio-demographic, environmental and behavioural factors among under-five children.

**Results:**

Children aged 6–11 months (AOR:3.5; 95% CI: 2.58–4.87), 12–23 months (AOR: 3.1; 95% CI: 2.33–4.04) and 24–35 months (AOR: 1.7; 95% CI: 1.26–2.34) as compared to > 35 months were significantly associated with an increasing prevalence of diarrhoea. Children in Afar region (AOR: 1.92; 95% CI: 1.01–3.64) and Gambela region (AOR: 2.12; 95% CI: 1.18, 3.81) were significantly associated with an increasing prevalence of diarrhoea, but a decreasing prevalence in Somali region (AOR: .42; 95% CI: (.217–.80) as compared to Tigray region. Increasing prevalence of diarrhoea was also significantly associated with male children (AOR: 1.3; 95% CI: 1.05–1.58); households who shared toilet facilities with other households (AOR: 1.4; 95% CI: 1.09–1.77); fourth birth order (AOR: 1.81; 95% CI: 1.17–2.79), and fifth and above birth order (AOR: 1.85; 95% CI: 1.22, 2.81) as compared to first order; and mother’s current age 35–49 years in a household with ≥3 under-five children (AOR: 4.7; 95% CI: 1.64–13.45) as compared to those maternal ages of 15–24 years in a household with ≤2 under-five children.

**Conclusion:**

The age of a child, sex of a child, region, birth order, toilet facilities shared with other households and the interaction effect of the number of under-five children with mother’s current age are identified as associated risk factors for diarrhoea occurrence among under-five children in rural Ethiopia. The findings show the need for planning and implementing appropriate prevention strategies considering these risk factors for rural under-five children.

## Background

According to World Health Organisation (WHO), diarrhoea is defined as the passage of three or more loose or liquid stools per day (or more frequent passage than is normal for the individual) [1]. Diarrhoea is usually a symptom of an infection in the intestinal tract, which can be caused by a variety of bacterial, viral and parasitic organisms. It can last several days and can leave the body without the water and salts that are necessary for survival. It depletes the body fluids and can cause severe dehydration, which can lead to death if not treated properly. Furthermore, severe dehydration and fluid loss are the direct causes of diarrhoeal death for most people [1].

Diarrhoea is a leading cause of malnutrition [2] and the second leading cause of death in children under 5 years old being responsible for killing around 525,000 children every year. Globally, there are nearly 1.7 billion cases of childhood diarrhoeal disease every year [1]. According to United Nations International Children’s Emergency Fund (UNICEF), 88% of all diarrhoeal deaths in 2015 were concentrated in South Asia and sub-Saharan Africa [2]. Low and lower-middle-income countries are home to 62% of the world’s under-five population but account for more than 90% of global pneumonia and diarrhoeal death [2]. In low-income countries, children under 3 years old experience on average three episodes of diarrhoea every year [1].

Ethiopia is one of the top 10 countries with the highest number of diarrhoeal deaths. Based on UNICEF report, 15,500 diarrhoeal deaths occurred among under-five children in Ethiopia in 2015 [2]. The 2005, 2011 and 2016 Ethiopian Demographic and Health Survey (EDHS) reports showed that the percentage of under-five children who had diarrhoea in the 2 weeks before the survey period were 18, 13 and 12%, respectively [3]. Even though the magnitude of diarrhoea have reduced over the past periods, diarrhoeal disease is still the major cause of morbidity and mortality among children in Ethiopia.

Studies conducted in different countries showed that rural children are more severely affected by diarrhoea than urban children [4–6]. The 2016 EDHS report showed that under-five children in rural Ethiopia had, relatively, more diarrhoea occurrence than urban ones [3]. Even though magnitude of diarrhoea disease in rural Ethiopia is high, there are limited pocket studies conducted at District/Zone/Town level to assess diarrhoeal disease and associated risk factors. Moreover, there is no study at the country/national level, which focused specifically on the rural part of the country, to show associated risk factors of diarrhoea occurrence among under-five children. So, evidence based information is needed for a child’s health improvement strategy by preventing and reducing the severity of diarrhoeal in under-five children in rural Ethiopia. Thus, this study was conducted to fill this gap by identifying the socio-demographic, environmental and behavioural associated risk factors of the occurrence of diarrhoea among children aged under 5 years in rural Ethiopia.

## Methods

### Study design and setting

This study was based on a national community-based cross-sectional study, EDHS 2016, in Ethiopia. The EDHS 2016 was conducted from January 18, 2016, to June 27, 2016. The survey was the fourth survey in the country. More details can be accessed from the EDHS 2016 report [3].

### Sampling design and data

The sampling frame used for the 2016 EDHS was the 2007 Ethiopian Population and Housing Census frame, which was provided by the Central Statistical Agency (CSA). The 2016 EDHS samples were selected using two stages stratified sampling procedure. Each region was stratified into urban and rural areas, which yielded 21 (11 urban and 10 rural) sampling strata. Samples of enumeration areas (EAs) were selected independently in each stratum in two stages. In 2016 EDHS, a sample of 10,641 under-five children represented by interviewed mothers were included. Out of these children, a complete response about the two-week occurrence of diarrhoea was obtained for 9916. Among them, 1875 children were from urban residences. Hence, after children with no response about the diarrhoea case and those from urban parts of Ethiopia being excluded, 8041 under-five children with complete information were used as the data for this study.

Thus, for this study, all under-five children in rural parts of Ethiopia were extracted from the EDHS 2016 data. EDHS 2016 was retrieved from major DHS after describing the objective of the study.

### Study variables

The response variable was the reported occurrence of diarrhoea. The mother was asked if the child had diarrhoea in the last 2 weeks.

The independent variables included in the study were identified from literature conducted earlier and associated with the occurrence of diarrhoea among under-five children [4, 7–13]. These are the socio-demographic variables (current age of mother, sex of child, current age of child, child lives with whom, region, mother educational level, religion, birth order, work status of the mother, household wealth index, number of under-five children in the household, current marital status of the mother, current breastfeeding status and number of household members); environmental and behavioural variables (source of drinking water, type of toilet facility, if toilet facility is shared with other households, disposal of youngest child’s stools when not using toilet and main floor material).

### Data analysis methods

After the extracted data were checked for completeness and coded, the analyses were done using SPSS Version 23. Data were described and summarized through frequencies and percentages. To study the effect of the different independent variables on the response variable, bivariate and multivariable analysis were used. In bivariate analysis, chi-square test of association and crude odds ratio were estimated to assess the association between each of the independent variables and the response variable. The backward stepwise method was used to select variables for the best-reduced model and Wald-test was used to test individual significance of the coefficients of the model.

A multivariable binary logistic regression model was used to identify associated risk factors of diarrhoea occurrence among under-five children. The overall goodness of the final model was checked using the Hosmer-Lemeshow goodness-of-fit test. Interpretations of the strength of the associations between associated risk factors and the response variable were based on significant adjusted odds ratios (AOR) with their respective 95% confidence intervals at 5% level of significance (*p*-value < 0.05).

## Results

### Socio-demographic, environmental and behavioural characteristics of the study participants

The study included 8041 under-five children. Out of these 3938 (49.0%) were females, 891 (11.1%) were less than 6 months and 3265 (40.6%) were age greater than 35 months (Table 1). The majority of the children 5459 (67.9%) were currently breastfeeding at the time of the survey. Around 4774 (59.4%) of the children were from households with six or more family members. Regarding mothers, 5801 (72.1%) had no formal education and 7659 (95.2%) were married.

**Table 1:** Socio-demographic characteristics related to under-five children and their bivariate analysis with the occurrence of diarrhoea in rural Ethiopia

Variables		Had diarrhoea recently		χ^2^-test
	Counts (%)	No (%)	Yes (%)	*P*-value
**Current age of mother (year)**				**0.004**
15–24	1972(24.5)	1724 (87.4)	248 (12.6)	
25–34	4080 (50.7)	3610 (88.5)	470(11.5)	
35–49	1989(24.7)	1803(90.6)	186(9.4)	
**Sex of child**				**0.042**
Male	4103(51.0)	3613(88.1)	490(11.9)	
Female	3938(49.0)	3524(89.5)	414(10.5)	
**Current age of child**				**0.000**
< 6 month	891(11.1)	816(91.6)	75(8.4)	
6–11	801(10.0)	650(81.1)	151(18.9)	
12–23	1517(18.9)	1243(81.9)	274(18.1)	
24–35	1567(19.5)	1378(87.9)	189(12.1)	
> 35 month	3265(40.6)	3050(93.4)	215(6.6)	
**Education of mother**				**0.050**
No formal education	5801(72.1)	5182(89.3)	619(10.7)	
Primary	1912(23.8)	1667(87.2)	245(12.8)	
Secondary	270(3.4)	235(87.0)	35(13.0)	
Higher	58(0.7)	53(91.4)	5(8.6)	
**Region**				**0.000**
Tigray	820(10.2)	726(88.5)	94(11.5)	
Afar	879(10.9)	787(89.5)	92(10.5)	
Amhara	838(10.4)	725(86.5)	113(13.5)	
Oromia	1414(17.6)	1250(88.4)	164(11.6)	
Somali	1106(13.8)	1032(93.3)	74(6.7)	
Benishangul	765(9.5)	692(90.5)	73(9.5)	
SNNP	1104(13.7)	946(85.7)	158(14.3)	
Gambela	491(6.1)	431(87.8)	60(12.2)	
Harari	364(4.5)	320(87.9))	44(12.1)	
Dire Dawa	260(3.2)	228(87.7)	32(12.3)	
**Number of under 5 children in a household**				**0.001**
2 or less	6342(78.9)	5589(88.1)	753(11.9)	
3 and above	1699(21.1)	1548(91.1)	151(8.9)	
**Wealth index of the household**				**0.015**
Poor	5194(64.6)	4649(89.5)	545(10.5)	
Middle	1332(16.6)	1167(87.6)	165(12.4)	
Rich	1515(18.8)	1321(87.2)	194(12.8)	
**Birth order number**				0.185
1st order	1362(16.9)	1195(87.7)	167(12.3)	
2nd	1224(15.2)	1103(90.1)	121(9.9)	
3rd	1152(14.3)	1011(87.8)	141(12.2)	
4th	1062(13.2)	935(88.0)	127(12.0)	
5th and above	3241(40.3)	2893(89.3)	348(10.7)	
**Religion**				**0.011**
Orthodox	2108(26.20)	1859(88.2)	249(11.8)	
Catholic	46(0.6)	40(87.0)	6(13.0)	
Protestant	1492(18.6)	1291(86.5)	201(13.5)	
Muslin	4232(52.6)	3801(89.8)	431(10.2)	
Traditional/other	163(2.0)	146(89.6)	17(10.4)	
**Mother’s current work status**				**0.053**
Not working	6046(75.2)	5390(89.1)	656(10.9)	
Working	1995(24.8)	1747(87.6)	248(12.4)	
**Mother’s marital status**				0.242
Married/Living with partner	7659(95.2)	6805(88.8)	854(11.2)	
Widowed/separated/never in union	382(4.8)	332(86.9)	50(13.1)	
**Number of household members**				**0.046**
5 and fewer	3267(40.6)	2872(87.9)	395(12.1)	
6 and above	4774(59.4)	4265(89.3)	509(10.7)	
**Child lives with whom**				0.141
Mother	7904(98.30)	7010(88.7)	894(11.3)	
Other caregiver	137(1.70)	127(92.7)	10(7.3)	
**Currently breastfeeding**				**0.002**
No	2582(32.1)	2333(90.4)	249(9.6)	
Yes	5459(67.9)	4804(88.0)	655(12.0)	

χ^2^ Chi-square

Concerning the environmental and behavioural characteristics of the households, 3713 (46.7%) of mothers/caregivers have used unimproved source of drinking water (Table 2). There were 3262 (41.0%) unimproved toilet facility and 4103 (51.6%) no toilet facility in the household. Furthermore, around 3171 (61.0%) of the mothers/caregivers were not properly discarding the youngest child’s stools.

**Table 2:** Environmental and behavioural characteristics related to under-five children and their bivariate analysis with the occurrence of diarrhoea in rural Ethiopia

Variables		Had Diarrhoea recently		χ^2^-test
	Counts (%)	No (%)	Yes (%)	*P*-value
**Source of drinking water**				0.689
Improved water	4241(53.3)	3763(88.7)	478(11.3)	
Unimproved	3713(46.7)	3305(89.0)	408(11.0)	
**Type of toilet facility**				**0.034**
Improved toilet facility	589(7.4)	530(90.0)	59(10.0)	
Unimproved toilet facility	3262(41.0)	2863(87.8)	399(12.2)	
No Facility/bush/field	4103(51.6)	3675(89.6)	428(10.4)	
**Toilet facilities shared with other household**				**0.041**
No	2986(77.5)	2648(88.7)	338(11.3)	
Yes	865(22.5)	745(86.1)	120(13.9)	
**Disposal of youngest child’s stools**				**0.070**
Proper disposal	2013(38.8)	1754(87.1)	259(12.9)	
Improper disposal	3171(61.2)	2816(88.8)	355(11.2)	
**Main floor material**				
Natural floor	7413(93.2)	6594(89.0)	819(11.0)	0.634
Rudimentary floor	72(0.9)	63(87.5)	9(12.5)	
Finished floor	469(5.9)	411(87.6)	58(12.4)	

χ^2^ Chi-square

### Prevalence of Diarrhoea

**Fig. 1:**
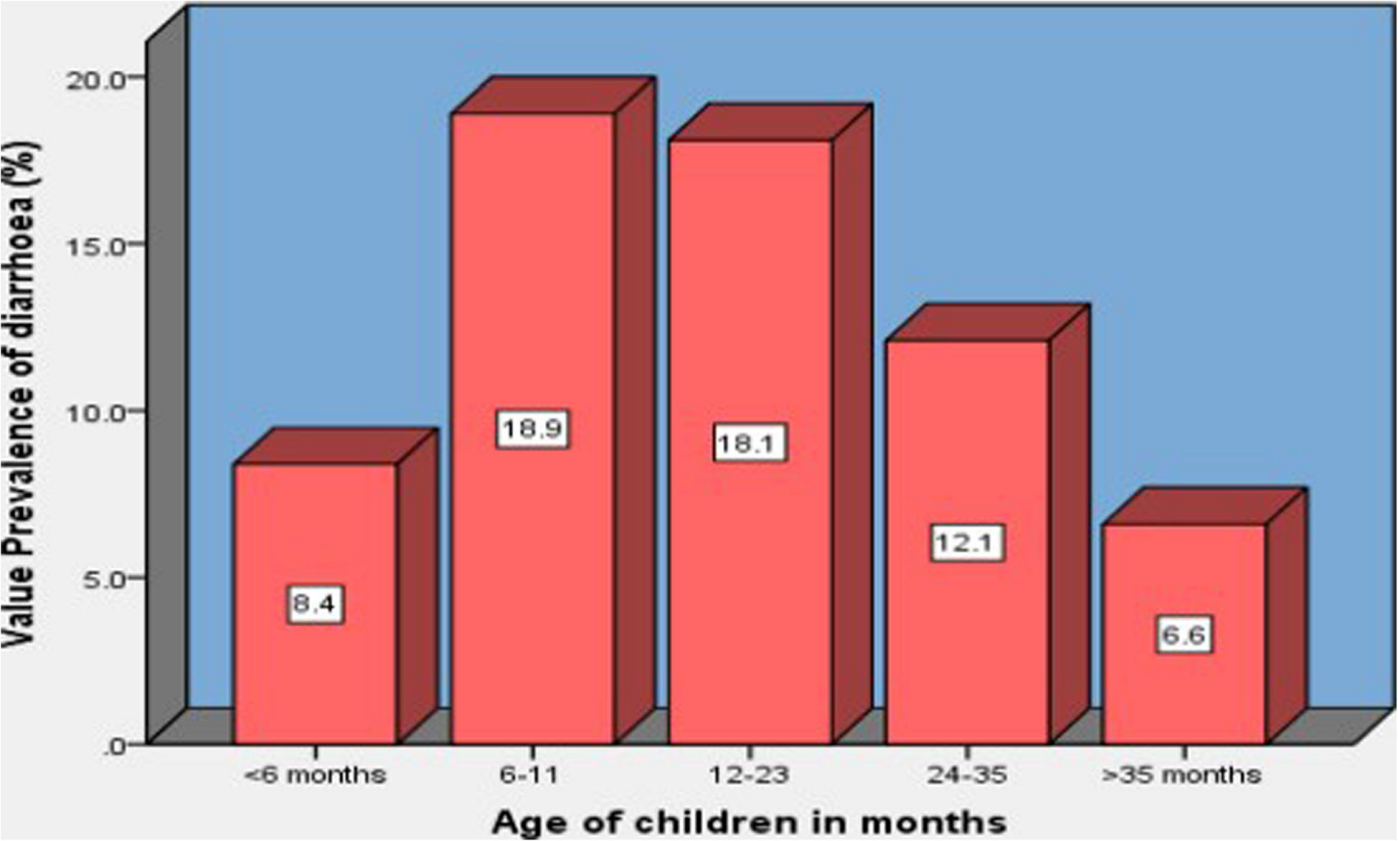
Age-specific prevalence of diarrhoea among under-five children. The heights of the bars show the prevalence of diarrhoea highly increases among children age from 6 months to 11 months, and then gradually decreases to age above 35 months

The two-week prevalence of diarrhoea among under-five children was 11.2% (95% CI: 10.5–11.9%) in rural Ethiopia. The result displayed in Tables 1 and 2 showed that the occurrence of diarrhoea was highest among children age 6–11 months (18.9%) and 12–23 months (18.1%). For ease of comparison, visual display for the prevalence of diarrhoea by age among children is also given (Fig. 1]. The highest prevalence of diarrhoea was also observed in children living in southern Nations Nationalities and People’s region (SNNPR) (14.3%), whose household shared toilet facility with other households (13.9%), protestant followers’ children (13.5%) and widowed/separated mothers’ children (13.1%). Tables 1 and 2 also show that there are other socio-demographic, environmental and behavioural characteristics of the children that the occurrence of diarrhoea was above the overall average (11.2%).

### Associated risk factors of Diarrhoea

In bivariate analysis, the chi-square test results (Tables 1 and 2) and the estimated crude odds ratios (Table 3) showed that there were a significant association between occurrence of diarrhoea and mother’s current age, sex of a child, current age of a child, region, number of under-five children, wealth index of the household, religion, number of household members, current breastfeeding status, type of toilet facility and toilet facilities shared with other households at 5% level of significance.

**Table 3:** Multivariable binary logistic regression analysis of the effects of socio-demographic and environmental associated risk factors of occurrence of diarrhoea among under-five children in rural Ethiopia

Variables	COR(95% CI)	AOR(95% CI)	*P*-value for AOR
**Current age of mother**			
[15–24]	–	–	–
25–34	0.90(0.77, 1.07)	0.66(0.47, 0.93	**0.016**
35–49	0.72(0.59, 0.88)**	0.36(0.23, 0.58)	**0.000**
**Sex of child**			
(Female)	–	–	–
Male	1.15(1.00, 1.33)*	1.29(1.05, 1.58)	**0.013**
**Current age of child**			
[> 35 month]	–	–	–
< 6 month	1.30(0.99, 1.71)	1.34(0.91, 1.99)	0.140
6–11	3.30(2.63, 4.13)**	3.54(2.58, 4.87)	**0.000**
12–23	3.13(2.59, 3.78)**	3.07(2.33, 4.04)	**0.000**
24–35	1.95(1.58, 2.39)**	1.72(1.26, 2.34)	**0.001**
**Region**			
[Tigray]	–	–	–
Afar	0.90(0.67, 1.22)	1.91(1.01, 3.64)	**0.047**
Amhara	1.20(0.90, 1.61)	1.51(0.93, 2.46)	0.095
Oromia	1.01(0.77, 1.33)	1.24(0.80, 1.91)	0.338
Somali	0.55(0.40, 0.76)**	0.42(0.22, 0.80)	**0.009**
Benishangul	0.81(0.59, 1.12)	0.95(0.60, 1.52)	0.844
SNNP	1.290(.98, 1.69)	1.39(.90, 2.15)	0.134
Gambela	1.07(.76, 1.52)	2.12(1.18, 3.81)	**0.012**
Harari	1.06(.72, 1.55)	0.89(0.49, 1.62)	0.698
Dire Dawa	1.08(.71, 1.66)	0.72(0.31, 1.65)	0.440
**Birth order number**			
[1st order]	–	–	–
2nd	0.78(0.61, 1.01)	0.91(0.62, 1.34)	0.647
3rd	0.99(0.79, 1.27)	1.48(0.98, 2.22)	0.059
4th	0.97(0.76, 1.24)	1.81(1.17, 2.79)	**0.007**
5th and above	0.86(0.71, 1.05)	1.85(1.22, 2.81)	**0.004**
**Toilet facilities shared with other households**			
[No]	–	–	–
Yes	1.26(1.01, 1.58)*	1.39(1.09, 1.77)	**0.008**
No. of under 5 children*Age of mother			
[(2 or less)*(15–24)]		–	–
NoChildU5(≥3) by ageM(25–34)		2.46(0.96, 6.30)	0.060
NoChildU5(≥3) by ageM(35–49)		4.70(1.64, 13.45)	**0.004**

The reference categories are those indicated in square brackets

*Statistically significant variables at *p* < 0.05; **statistically significant variables at *p* < 0.01

In multivariable analysis, the Hosmer-Lemeshow goodness-of-fit test result (*P*-value = 0.763) showed that the final multivariable binary logistic regression model was a good fit to the data.

The result showed that current age of child [6–11 months (AOR: 3.5; 95% CI: 2.58–4.87), 12–23 months (AOR: 3.1; 95% CI: 2.33–4.04) and 24–35 months (AOR: 1.7; 95% CI: 1.26–2.34) as compared to > 35 months], sex of child [male (AOR: 1.3; 95% CI: 1.05–1.58)], region [Afar (AOR: 1.92; 95% CI: 1.01–3.64), Somali (AOR: 0.42; 95% CI: (0.217–0.80) and Gambela (AOR: 2.12; 95% CI: 1.18–3.81) as compared to Tigray region], birth order [4th (AOR: 1.81; 95% CI: 1.17–2.79), 5th and above (AOR: 1.85; 95% CI: 1.22–2.81) as compared to 1st order], toilet facilities shared with other households (AOR: 1.4; 95% CI: 1.09–1.77) and mother’s current age 35–49 years in a household with three or more under-five children (AOR: 4.7; 95% CI: 1.64–13.45) as compared to those maternal age of 15–24 years in a household with less than or equal to two under-five children were statistically significant associated risk factors of diarrhoeal occurrence among under five children at 5% level of significance (Table 3).

## Discussion

This study was intended to identify demographic, environmental and behavioural associated risk factors of the occurrence of diarrhoea among under-five children in rural Ethiopia based on 2016 EDHS data.

In this study, the variables current age of a child, sex of a child, region, birth order, toilet facilities shared with other households and the interaction effect of number of under-five children with age of mother were identified as associated risk factors for under-five diarrhoeal disease occurrence.

The result indicated that child’s age group 6–11, 12–23 and 24–35 months were 3.5, 3.1 and 1.7 times more affected by diarrhoea than child’s age greater than 35 months respectively after adjusting for the effect of other variables. In general, children age greater than 35 months had a lower risk of having diarrhoea than children whose age between 6 and 35 months. This may be due to the fact that children whose age between 6 and 23 months begin supplementary foods and also they start crawling and can touch contaminated materials in unclean environment and immediately return their hand to mouth, so this may cause them to be easily exposed to diarrhoeal disease. The 2016 EDHS also reported that diarrhoea prevalence remains high (18%) at age of 12–23 months, which is the time when children begin walking and are at increased risk of contamination from the environment [3]. Recent studies and scientific knowledge also show that a lot of diarrhoea in this age is due to rotavirus. For instance, a study conducted in Farta Woreda, North West Ethiopia showed that children age 6–11 months and not vaccinated for rotavirus are highly affected by diarrhoea [14, 15]. A study in Burkina Faso also showed that rotavirus is more prevalent in young children (< 12 months) and children less than 12 months of age were susceptible to diarrhoea [16]. Therefore, the role of rotavirus vaccines is also important. Moreover, increased risk of disease in younger children might be due to naive immune system in children of younger ages and waning of maternal antibodies [16–21]. The finding of this study is also in line with studies done in Benishangul region, Eastern Ethiopia, Enderta Woreda, Wolitta Soddo and southern Ethiopia [4, 7, 22–25].

Sex of a child had a significant association with diarrhoea occurrence. Male children were 1.3 times more affected by diarrhoea than female children after adjusting for the effect of other variables. A study conducted in Dhaka, Bangladesh in 2018 similarly concluded that more boys presented with acute diarrhoeal illness than girls [26]. This might be because of possibility of a sex-based difference in the pathophysiology of acute pediatric diarrhoea that we do not yet understand [26].

The study also revealed that the occurrence of diarrhoea was significantly associated with region of the mother. Children in rural Afar and rural Gambela regions were twice as likely to be affected by diarrhoea as compared to children in rural Tigray region. However, under-five children from the rural Somali region were 0.45 times less likely to be affected by diarrhoea than those from rural Tigray region.

Households those shared toilet facility with other households had a significant association with diarrhoeal disease. Children from households with shared toilet facility had around 39% more risk for having diarrhoea than those from households who did not share toilet facilities. Thus, children under the age of five face an increased risk of contracting diarrhoea when they share a toilet with just one or two other households. Epidemiological studies have identified an increased risk of diarrhoeal diseases associated with using shared sanitation facilities. A similar study conducted using data from 51 countries’ demographic and health surveys found that shared sanitation appears to be a risk factor for diarrhoea although differences in socioeconomic status are important [27]. An analytical review study conducted by Ramlal et al. in 2019 also found that the use of shared sanitation showed a significant increase in diarrhoeal disease, with an overall OR of 2.39 (85% CI 1.15–8.31) [28].

Children whose birth order 4th and 5th and above were around 1.8 times more likely to be affected by diarrhoea than 1st order children. This result was in line with the findings in the Benishangul Gumuz region [7] and in Jigjiga district, Somali region [29].

The effect of number of under-five children in the household on childhood diarrhoea varies by current age of mothers. Number of under-five children affects the occurrence of diarrhoea differently in older age mothers (35–49) versus younger age mothers (15–24). The odds of the occurrence of diarrhoea for three or more number of under-five children (relative to two or less number of under-five children) in older age mothers was 4.7 times as high as in younger age mothers. In other words, mother’s current age 35–49 years in a household with three or more under-five children had significantly greater prevalence of diarrhoea as compared to those maternal age of 15–24 years in a household less than or equal to 2 under-five children. Older mothers, on average, can have a higher number of children as compared to younger ones. As a result, it may be difficult to give care effectively when the number of under-five children becomes large in a household and then it may be a cause for the occurrence of diarrhoea. Therefore, to control the number of under-five children the role of family planning is important. Effective family planning can also reduce the number of high birth ordered children in the household. This finding is consistent with a study done in eastern Ethiopia [4], the Benishangul Gumuz region [7], northeast Ethiopia [30] and in northwest Tigray [31].

### Limitations

In this study, possible modifiable risk factors like rotavirus vaccine, hand washing, malnutrition status, and others were not included as they were captured with high missing values in the secondary data used for this study. The readers are requested to take this into account.

## Conclusion

The age of a child, sex of a child, region, birth order, toilet facilities shared with other households and the interaction effect of number of under-five children with current age of mothers are identified as associated risk factors for diarrhoea occurrence among under-five children in rural Ethiopia. The findings show the need for planning and implementing appropriate prevention strategies considering the identified risk factors that target rural under-five children. For instance, strategies for reducing the number of under-five children and birth order in the household as well as supportive strategies about household sanitation facilities (toilet facility and its usage), and women education on appropriate child care practices tailored by their age might reduce prevalence of diarrhoea. Further research is recommended to investigate the determinants of diarrhoea using primary data including all modifiable associated risk factors like rotavirus vaccine, hand washing, malnutrition status, and others in rural Ethiopia.

## Data Availability

The general datasets are available from the Central Statistical Agency and the DHS Program data home, USAID. Specifically, the minimal data used for this study are available from the corresponding author on reasonable request.

## Abbreviations

AOR: Adjusted Odds Ratio
CI: Confidence interval
COR: Crude odds Ratio
CSA: Central Statistical Agency
DHS: Demographic and Health Survey
EAs: Enumeration Areas
EDHS: Ethiopia Demographic and Health Survey
SNNPR: Southern Nations, Nationalities, and People’s Region
UNICEF: United Nations International Children’s Emergency Fund
